# CRISPR/Cas9: A Tool to Circumscribe Cotton Leaf Curl Disease

**DOI:** 10.3389/fpls.2016.00475

**Published:** 2016-04-12

**Authors:** Zafar Iqbal, Muhammad N. Sattar, Muhammad Shafiq

**Affiliations:** ^1^Institute of Biochemistry and Biotechnology, Quaid-i-Azam Campus, University of the PunjabLahore, Pakistan; ^2^Department of Environment and Natural Resources, Faculty of Agriculture and Food Science, King Faisal UniversityAl-Hasa, Saudi Arabia; ^3^Agricultural Biotechnology Division, National Institute for Biotechnology and Genetic EngineeringFaisalabad, Pakistan

**Keywords:** begomoviruses, alphasatellite, betasatellites, cotton leaf curl disease, CRISPR/Cas9

## Abstract

The begomoviruses (family *Geminiviridae*) associated with cotton leaf curl disease (CLCuD) pose a major threat to cotton productivity in South–East Asia including Pakistan and India. These viruses have single-stranded, circular DNA genome, of ∼2800 nt in size, encapsidated in twinned icosa-hedera, transmitted by ubiquitous whitefly and are associated with satellite molecules referred to as alpha- and betasatellite. To circumvent the proliferation of these viruses numerous techniques, ranging from conventional breeding to molecular approaches have been applied. Such devised strategies worked perfectly well for a short time period and then viruses relapse due to various reasons including multiple infections, where related viruses synergistically interact with each other, virus proliferation and evolution. Another shortcoming is, until now, that all molecular biology approaches are devised to control only helper begomoviruses but not to control associated satellites. Despite the fact that satellites could add various functions to helper begomoviruses, they remain ignored. Such conditions necessitate a very comprehensive technique that can offer best controlling strategy not only against helper begomoviruses but also their associated DNA-satellites. In the current scenario clustered regulatory interspaced short palindromic repeats (CRISPR)/CRISPR associated nuclease 9 (Cas9) has proved to be versatile technique that has very recently been deployed successfully to control different geminiviruses. The CRISPR/Cas9 system has been proved to be a comprehensive technique to control different geminiviruses, however, like previously used techniques, only a single virus is targeted and hitherto it has not been deployed to control begomovirus complexes associated with DNA-satellites. Here in this article, we proposed an inimitable, unique, and broad spectrum controlling method based on multiplexed CRISPR/Cas9 system where a cassette of sgRNA is designed to target not only the whole CLCuD-associated begomovirus complex but also the associated satellite molecules.

## Introduction

**FIGURE 1:**
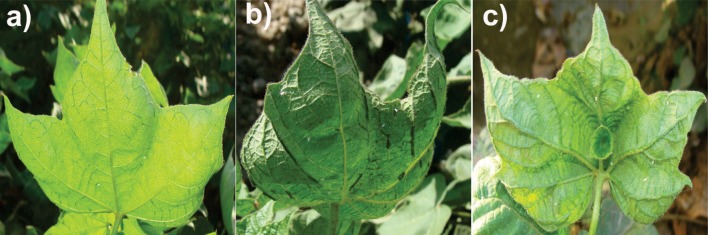
**Cotton (*Gossypium hirsutum* L.) plants showing typical symptoms of CLCuD caused by CAB (s) alone **(a)**, associated with alpha- and betasatellite **(b,c)**, respectively.** Presence of betasatellite incurred severe vein thickening and leaf enations on the underside of the leaves.

**FIGURE 2:**
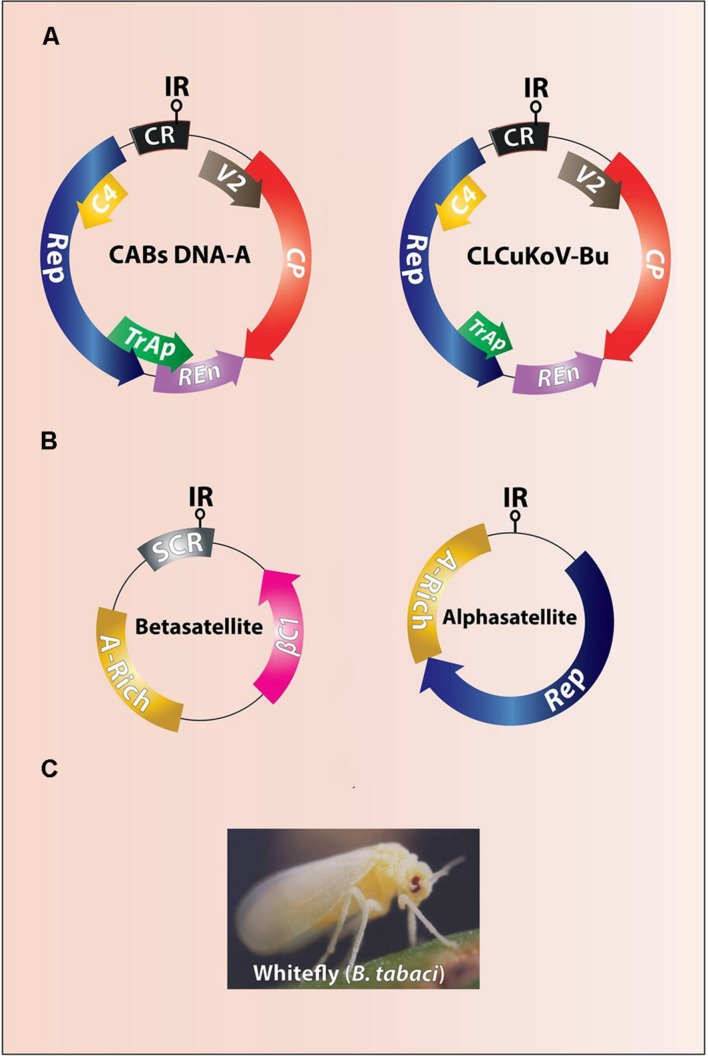
**Genome organization of CABs (A) and associated DNA-satellites (B).** The difference between genomes of resistance breaking CLCuKoV-Bu and other CABs is of a truncated *TrAP* **(A)**. The intergenic region (IR) contains stem loop structure and the origin of replication (ori). The CABs and associated DNA-satellites are vectored by ubiquitous whitefly (*Bemisia tabaci*) **(C)**.

Cotton leaf curl disease (CLCuD) is a top ranked endemic disease to cotton in Pakistan, northwestern India and Africa, and causes a severe short fall in the economy thus, it is detrimental to the socio-economic values of the people (Mansoor et al., 2003; Varma and Malathi, 2003; Sattar et al., 2013). CLCuD on the Indian subcontinent is caused by a complex of begomoviruses in association with certain satellite molecules (alpha- and betasatellite). Viruses included in this complex are *Cotton leaf curl Alabad Virus* (CLCuAlV), *Cotton leaf curl Bangalore virus* (CLCuBaV), *Cotton leaf curl Kokhran virus* (CLCuKoV), CLCuKoV-Bu (Burewala strain), *Cotton leaf curl Multan virus* (CLCuMuV), and *Cotton leaf curl Rajasthan virus* (CLCuRaV) (Zhou et al., 1998; Kirthi et al., 2004; Yousaf et al., 2013). All CLCuD-associated begomoviruses (CABs) induce typical symptoms of leaf curling (upward and downward), vein swelling, enation, and stunted growth (**Figure 1**). CABs belonging to genus begomovirus (Family *Geminiviridae*) are exclusively transmitted by ubiquitous whitefly (*Bemisia tabaci*) and have a circular single-stranded DNA genome of ∼2800 nt in size (**Figure 2A**) encapsidated in twinned quasi-icosahedera (Briddon and Markham, 2001; Böttcher et al., 2004). The genome of CABs encodes six genes in bi-directional manner separated by non-coding intergenic regions (IR), containing promoter elements and the origin of replication (**Figure 2A**). Two virion sense genes, the coat protein (*CP*) and *V2* (pre-coat protein), are involved in insect transmission, encapsidation, *in planta* movement and suppression of host defense (Rojas et al., 2001). The remaining four complementary sense genes include the replication-associated protein [(*Rep*; sole protein involved in viral replication; (Hanley-Bowdoin et al., 2004)], the *C2/TrAP* protein [(transcriptional activator protein; involved in suppression of host defense, activation of late genes and overcomes virus induced hypersensitive cell death; Hussain et al., 2007; Yang et al., 2007; Mubin et al., 2010)], the replication enhancer protein *C3*/*REn*; (Settlage et al., 2005) and the *C4* protein [(a pathogenicity/symptoms determinant and a suppressor of PTGS; Vanitharani et al., 2004; Saeed et al., 2008; Iqbal et al., 2012; Sunitha et al., 2013)]. Contrary to helper begomovirus, beta- and alphsatellites are half the size (∼1350 nt) of their helper begomoviruses, each encoding a single protein β*C1* and *Rep*, respectively (Mansoor et al., 2001; Briddon et al., 2004) (**Figure 2B**). Of these two DNA-satellites, betasatellites are more diverse and their encoded single protein, β*C1*, adds more functions towards the viral pathogenesis than the alphasatellites. This protein is a suppressor of host defense, a pathogenicity/symptom determinant (Saeed et al., 2005; Qazi et al., 2007), may be involved in movement (Saeed et al., 2007), increases the helper begomovirus titer (Briddon et al., 2001), complements the missing functions of helper begomovirus genes (Iqbal et al., 2012), modulates the levels of developmental microRNAs (Amin et al., 2011), binds to DNA/RNA in sequence independent manner (Cui et al., 2005), interacts with various host-encoded factors, forms multimers, and also interacts with *CP* (Cheng et al., 2011), and suppresses jasmonic acid production in plants (Zhang et al., 2012).

Although the exact history of CLCuD is difficult to map, CLCuD was, however, established in the sub-continent due to introgression of high yielding but highly susceptible varieties of cotton (*Gossypium hirsutum*) in place of local resistant cotton species (*G. arboreum*) (Ali, 1997). Symptoms of this disease were discerned in 1967 near Multan, Pakistan (Hussain and Ali, 1975). Later in 1980, its first outbreak linked with dramatic decrease in cotton productivity gave scientists and policy makers cause for serious concern. In 1992–1997, it surged again and about 0.2 million hectares (mha) out of 2.5 mha were affected resulting in 29% reduction in cotton productivity and the country had to suffer a short fall in economy of about $US 5 billion (Briddon and Markham, 2001). Subsequently, CLCuD spread across the borders and reported from Sriganganagar, India in 1993 (Kumar et al., 2010). In the last decade of the previous century, continuous conventional breeding efforts and introduction of resistant/tolerant cotton varieties in Pakistan heaved the economy. However, in 2001–2004, the second outbreak was observed and the disease spread to southern parts of Pakistan, the Sindh province. Later, investigations showed that this outbreak was associated with a recombinant begomovirus, *Cotton leaf curl Burewala virus* associated with recombinant Cotton leaf curl Multan betasatellite (CLCuMuB) (Briddon, 2003; Mahmood et al., 2003). *Cotton leaf curl Burewala virus*, now referred as CLCuKoV-Bu (Brown et al., 2015), has also been established widely in India (Rajagopalan et al., 2012; Zaffalon et al., 2012). Recently, different variants of CLCuKoV-Bu have been identified from Pakistan and India (Shuja et al., 2014; Kumar et al., 2015), hence posing the possibility of another alarming situation that can lead to the third outbreak in sub-continent. Until now, despite all efforts (both conventional and molecular approaches) the almost the whole available germplasm is susceptible, except few lines which are claimed to be tolerant, against this menace (Rahman and Zafar, 2007).

The failure of controlling strategies against these begomoviruses has urged the scientists to explore better measures to control CLCuD. In the current scenario, CRISPR/Cas9, as an extremely versatile, multifaceted and emerging technology, has gained the attention of research communities across the globe. This system has recently been exploited successfully against *Bean yellow dwarf virus* (BeYDV) (Baltes et al., 2015), *Beet severe curly top virus* (BSCTV) (Ji et al., 2015), and *Tomato yellow leaf curl virus* (TYLCV) (Ali et al., 2015), where reduced viral load up to 97% has been achieved linked to attenuated disease symptoms and apparently with no off-target affects. Keeping in mind the successful implementation of CRIPSR/Cas9, here in this study, a tentative methodology based on multiplex CRIPSR/Cas9 system is proposed, where universal sgRNAs are designed to target the most conserved regions of CABs and their associated DNA-satellite molecules that can potentially interfere with replication and movement of CABs and their cognate satellites, hence can resuscitate the cotton production by curbing CLCuD.

## Why CRIPSR/Cas9 Against CLCUD?

In the past few decades, CABs have proliferated extensively. Their evolution and dispersion may likely be exacerbated by recombination, selection pressure imposed by introgression of resistant cultivars, change in virus complex and rise in temperature due to global warming. Despite concerted efforts made against CLCuD (see Techniques Employed to Control Cabs), nothing has been entirely successful in yielding resistance to this disease (Farooq et al., 2011; Sattar et al., 2013). The prevalent situation of unfruitful controlling strategies against CLCuD necessitates devising more strict controlling mechanisms that can curtail the CABs and their associated satellites. Under such circumstances, CRISPR/Cas9 system offers substantial advantage. The CRISPR/Cas9 system, apart from Zinc finger (ZFN) and Transcription activator-like effector nucleases (TALENs), has gained the attention of scientists across all major fields of science, especially plant biologists, as a promising programmable genome editing tool with higher level of specificity. Moreover, robustness, wide adaptability, and easy engineering of this system have proved its potential as a tool to control viruses – especially CABs. Very recently, this has also been successfully used to control plant viruses including different geminiviruses (Ali et al., 2015; Baltes et al., 2015; Ilardi and Tavazza, 2015; Ji et al., 2015). At the advent of CRISPR/Cas9 system, the major limitation observed was the degree to which off-target mutations take place. Until now, only few studies showed very negligible off-target activities. Improved versions of CRISPR/Cas9 DNA editing system and bioinformatics tools (**Table 1**) have increased the specificity to next level. New versions of sgRNAs can direct endonuclease Cas9 to induce precise cleavage at a target site (Jinek et al., 2012). In short, this system has validated the capability across a wide range of living organism to accomplish gene interference, where it has demonstrated its applicability and efficacy, thus rendering it as a final choice to curb viral diseases (Feng et al., 2013; Li et al., 2013), especially against CABs.

**Table 1:** Available bioinformatics tools for selecting optimal CRISPR/Cas9 target sites and predicting off-targets.

Purpose	Web address	Reference
CRISPR/Cas9 design tool to find target sites within an input sequence.	http://www.genome-engineering.org	Hsu et al., 2013
Designed sgRNA can be checked for off-targets and specificity against different genomes including *Arabidopsis thaliana*.	http://crispr.mit.edu/	Ran et al., 2013
Online tool for designing highly active sgRNAs.	http://www.broadinstitute.org/mai/public/analysis-tools/sgma-design	Doench et al., 2014
Open sourced tool that is used locally, designed to identify potential off-target sites in any user specified genome.	http://eendb.zfgenetics.org/casot/	Xiao et al., 2014
Download link to access 38 plant genomes.	http://plants.ensembl.org/info/website/ftp/index.html	
Web-based tool to design sgRNA sequences for genome library projects or individual sequences. Target site homology is also evaluated to predict off targets. Five plant genomes are available.	http://www.e-crisp.org/E-CRISP/designcrispr.html	Heigwer et al., 2014
Eight representative plant genomes are available to predict sgRNAs with low chance of off-target sites.	www.genome.arizona.edu/crispr	Xie et al., 2014
Online tool for accurate target sequence selection and prediction of off-target binding of sgRNAs. Includes the design of target specific primers for PCR genotyping. The only plant genome available is *A. thaliana*.	https://chopchop.rc.fas.harvard.edu/	Montague et al., 2014
Cas-OFFinder.	http://www.rgenome.net/cas-offinder/	Kleinstiver et al., 2016
Addgene service of non-profit plasmid distribution.	http://www.addgene.org/crispr/church/	Mali et al., 2013 Kleinstiver et al., 2016
	http://www.addgene.org/crispr-cas
Software for the analysis of GUIDE-seq.	http://www.jounglab.org/guideseq	Kleinstiver et al., 2016
Database that identifies Cas proteins and CRISPRs; includes many features complementary to CRISPR.	http://crispi.genouest.org/	Bhaya et al., 2011
CRISPR database; includes several tools to identify and analyze CRISPRs. Maintained by Universite Paris Sud.	http://crispr.upsud.fr/	Bhaya et al., 2011
To obtain Cas genes sequences.	http://mbgd.genome.ad.jp	Bolotin et al., 2005
	http://ergo.integratedgenomics.com/ERGO
Cas9 online designer.	http://cas9.wicp.net/	Wang et al., 2016


## Techniques Employed to Control CABs

A diverse group of techniques ranging from conventional breeding to advance molecular approaches have been implicated to control CLCuD, however, over time, resistance or tolerance was broken down by the virus (Aragão and Faria, 2009). Such techniques include accustom breeding approaches (Siddig, 1970; Ali, 1997; Rahman, 1997; Iqbal et al., 2001; Ahmad et al., 2010, 2011; Akhtar et al., 2010), pathogen derived resistance (PDR) including protein and non-protein mediated resistance (Amudha et al., 2010, 2011; Yousaf et al., 2013) (Asad et al., 2003; Sanjaya et al., 2005; Mubin et al., 2011; Ali et al., 2013; Tahir, 2014), DNA interference (Liu et al., 1998; Tahir, 2014), and non-PDR including expression of *cry1Ac* and *GroEL* (Akad et al., 2007; Edelbaum et al., 2009; Shepherd et al., 2009; Rana et al., 2012). Moreover, controlling the whitefly vector has also become a major focus of world renowned laboratories to control CLCuD through RNAi (author’s personal communication). These techniques are lagging in “fair control” of CLCuD. All of these techniques were deployed directly or indirectly to control only CABs but not their cognate DNA-satellites. The role of CABs associated DNA-satellites to salvage CABs has been totally ignored during course of these efforts. This scenario has put the scientist in a situation where they plan to deploy more than one strategies (pyramiding) at once, which itself is a very laborious job.

## Successful Implications of CRISPR/Cas9 System Against Geminiviruses

CRISPR/Cas9 systems have superiority over other nucleases such as ZFN and TALEN for targeting multiple genes at the same time. There is variety of multiplex models available for genome engineering using CRISPR/Cas9 system. Either these models involve single RNA Pol-III promotor (Guo et al., 2015) or several different promotors at the same time (Kabadi et al., 2014; Sakuma et al., 2014; Cobb et al., 2015) to drive sgRNA/s co-expression using a single expression vector. Using multiple promoters for sets of sgRNAs in a single vector is very laborious and inconvenient (Guo et al., 2015). The ultimate strategy is to use a single promoter to drive multiple sgRNAs with each of them following a direct repeat (DR) sequence (Cong et al., 2013; Jiang et al., 2013; Guo et al., 2015), which is very similar to the native strategy in *Streptococcus thermophilus* involving crRNA interspacers followed by almost similar sized conserved DR sequences (Guo et al., 2015).

The CRISPR/Cas9 system has been successfully employed in controlling BeYDV (Baltes et al., 2015), BSCTV (Ji et al., 2015), and TYLCV (Ali et al., 2015). Ji et al. (2015) found that viral load was significantly reduced (up to 97%) using different BSCTV constructs in *Nicotiana benthamiana* and *Arabidopsis thaliana* plants. Whereas, Baltes et al. (2015) reported 87% reduction in targeted viral load in *N. benthamiana* using different sgRNAs from BeYDV, which was further improved by a combination of two sgRNAs together. The strategy involving use of CRISPR/Cas9 mediated resistance against geminiviruses was further extended to TYLCV – a monopartite begomovirus causing yellow leaf curl disease in tomatoes in most of the tomato growing areas of the world (Ali et al., 2015). During their study Ali et al. (2015) found that sgRNA constructs targeting stem loop structure in IR conferred better resistance against TYLCV in *N. benthamiana* plants. These successful implications suggest that there is huge potential in employing CRISPR/Cas9 system against geminiviruses, thereby targeting several sites in the virus genome (Zlotorynski, 2015).

## Proposed Methodology for Designing Multiplex sgRNA to Circumvent CLCUD

**FIGURE 3:**
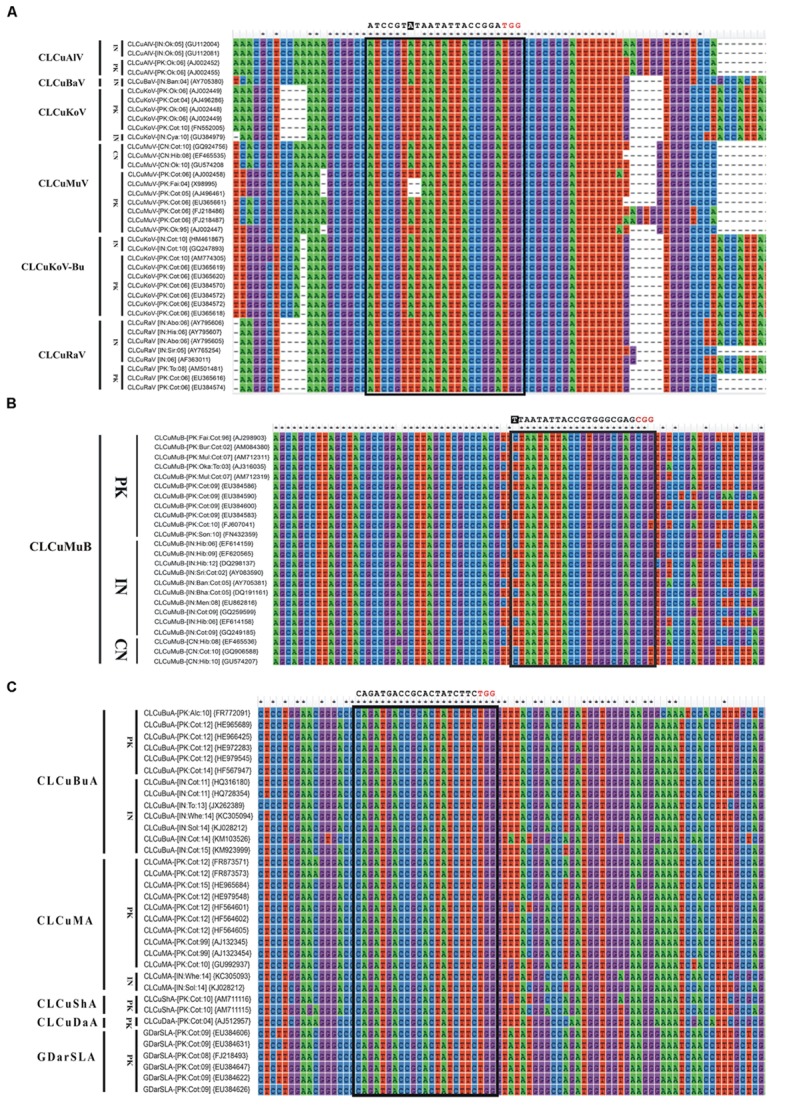
**Nucleotide sequence alignments of CABs **(A)** and their associated DNA-satellites **(B,C)**.** All the sequences were retrieved from NCBI GenBank database and aligned using ClustalW in Mega6. The isolate descriptors and accession numbers are given on the left side of each alignment. All the possible sgRNAs are summarized in the Supplementary Table S1. Only the recommended sgRNA sequence is given at the top of each alignment as black text. The protospacer adjacent motif (PAM) sequence is indicated in red text. Any mismatch outside of the seed sequence is highlighted as white text with black background.

**FIGURE 4:**
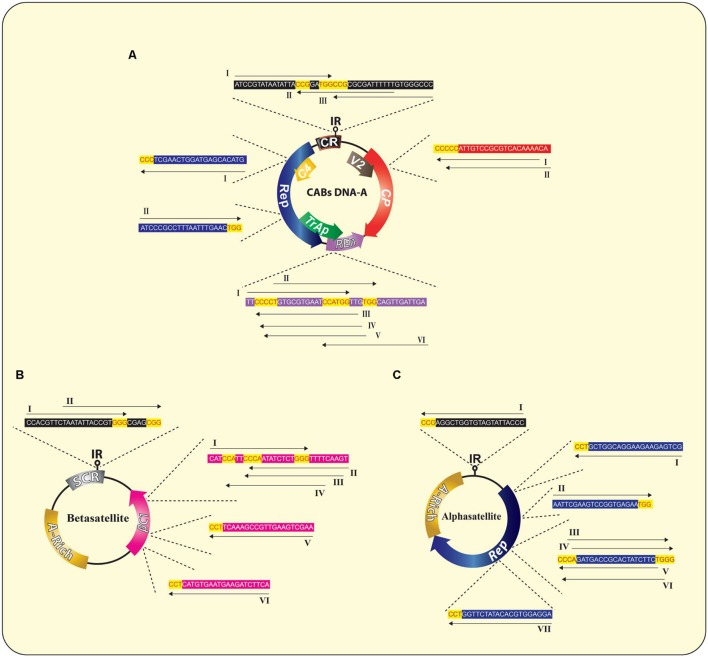
**Wild type genome of CABs **(A)** and associated DNA-satellites **(B,C)** illustrating the suggested sgRNAs sequences and their corresponding potential targets in each molecule, respectively.** The PAM sequences are indicated in red text with yellow background whereas, rest of the sequences are indicated in black text with colored background similar to the respective target gene. All sgRNAs are indicated as unbroken black arrows showing the direction of expression of each sgRNA. A detailed list of all possible sgRNAs is summarized in the Supplementary Table S1.

Till now, the CRISPR/Cas9 has been employed against just single species of geminiviruses, as previously mentioned, which limits this technology from reaching its full potential. To maximize the output of this technology, a multiplex type sgRNA could be designed that allow simultaneous editing of multiple begomoviruses in just one go and this is a noteworthy advantage of CRISPR/Cas9 system in comparison to other virus controlling strategies. For effective, rapid and wide-scale control over CABs, here we addressed key considerations for designing multiplex CRISPR/Cas9 system whose applications include, but not circumscribed to, simultaneous targeting of all types of CABs along with their cognate DNA-satellites (present in south east Asia). To achieve an absolute control over such a huge complex demands a multiplex genome editing strategy based on multiplex sgRNA that can potentially target whole CLCuD complex. For the said purpose, full length nucleotide sequences of CABs and their cognate DNA-satellites were retrieved from NCBI databank, aligned in Mega6 (Thompson et al., 1994) and most conserved regions, bearing protospacer adjacent motif (PAM) sequences, were selected to design multiplex sgRNA cassette (**Figures 3** and **4**). All the designed sgRNAs (16 for CABs, 08 for alphsatellites and 12 for CLCuMuB) were analyzed on the basis of available *Arabidopsis* genome for specificity, off-targets and quality score (Supplementary Table S1). Analyses showed that the majority of designed sgRNAs have off-target effects, though in non-coding regions, thus posing a major challenge in the course of their successful exploitation. Several strategies have been reported to enhance Cas9 specificity, including use of Cas9 nickase mutant (Mali et al., 2013; Ran et al., 2013), catalytically inactive Cas9 nucleases (Guilinger et al., 2014; Tsai et al., 2014), and few more strategies but with limited applications. Very recently, high-fidelity Cas9 nucleases were developed on the basis of structure-guided protein engineering strategy where a few amino acids were replaced successfully to reduce off-target effects and maintain robust on-target cleavage (Kleinstiver et al., 2016; Slaymaker et al., 2016). Importantly, Baltes et al. (2015) showed that designed sgRNA coupled with catalytically inactive Cas9 (dCas9) could control the BeYDV genome without cleaving the genome, hence, Cas9-HF (Kleinstiver et al., 2016; Slaymaker et al., 2016) or dCas9 (Baltes et al., 2015) could be used to eliminate the off-targets effects. We hereby proposed that same strategy could be employed here to reduce the off-target effects.

**FIGURE 5:**
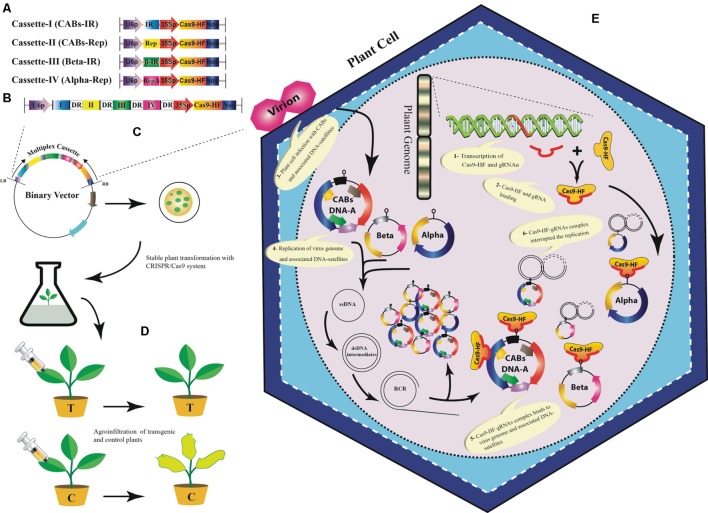
**A tentative approach to engineer resistance against CABs and their associated DNA-satellites using CRISPR/Cas9 system.** Graphical representation to construct sgRNA cassettes: A uniplex approach to construct individual sgRNAs for IR and Rep of CABs, βC1 of betasatellite and Rep-A of alphasatellite **(A)**, Multiplex approach to construct a joint cassette for IR and Rep (of CABs), βC1 and Rep-A to target CABs, betasatellite and alphasatellite, respectively, **(B)**. All of the sgRNAs are shown to be expressed from common RNA polymerase-III promoter (U6p). Each sgRNA is followed by a DR sequence (rounded rectangle) to ensure homologous recombination. Cas9 may be expressed from CaMV-35S promoter followed by a NOS terminator sequence. The multiplex cassette would be inserted between left- and right border of a suitable binary vector to carry out stable genetic transformation in *Nicotiana benthamiana* plants **(C)**. The control plants will be transformed with empty binary vector to ensure no resistance against CABs and associated DNA-satellites. The transgenic *N. benthamiana* plants will be agroinfiltrated with the CABs alone and/or with the associated DNA-satellites **(D)**. The transgenic plants successfully expressing CRISPR/Cas9 would show resistance against the viral complex whereas, the negative control plants will start showing typical CLCuD symptoms. A simultaneous *in planta* graphical representation of CRISPR/Cas9 system **(E)**: 1- Principle components of CRISPR/Cas9 system are transcribed from the plant genome in transgenic plants. 2- Assemblage of Cas9-HF and sgRNA. 3- Virus infection starts inside the cell after agroinfiltration using *Agrobacterium tumefacience*. 4- The single stranded DNA (ssDNA) of begomovirus and the associated DNA-satellites start proliferating by rolling circle replication (RCR) via a double stranded (ds) DNA intermediates in an infected plant cell. 5- The sgRNA and Cas9-HF complex bind at the complementary target sites along the dsDNA intermediates. 6- The activated sgRNA complex target the viral genome (s) and produce double-strand break (DSB) that can lead to degradation of viral and satellite genome. However, DSB can be repaired by non-homologous end joining (NHEJ) repair mechanism thus leaving the mutation in viral genome.

Our analyses showed that designed sgRNAs targeting IR regions have less off-targets in *Arabidopsis* genome (Supplementary Table S1). Moreover, it has been proved that sgRNAs targeting IR of geminiviruses confer better resistance as compared to other targeted sgRNAs in a geminivirus genome (Ali et al., 2015; Baltes et al., 2015). Therefore, we proposed a multiplex sgRNA cassette targeting IR and Rep of the CABs, IR of CLCuMuB and *Rep* of diverse alphasatellites, respectively (**Figures 5A,B**; Supplementary Table S1). Targeting IR could provide broad-spectrum resistance against these viruses under natural conditions because it is conserved among all members of the plant virus family *geminiviridae*, hence chance of resistance breaking would be minimized. To assemble such a cassette bearing multiple sgRNAs, many different strategies could be used such as golden gate cloning (Engler et al., 2008; Xing et al., 2014), array assembly (Guo et al., 2015), tRNA processing (Xie et al., 2015), and use of chimeric constructs (Cong et al., 2013). The expression of the gRNA cassette should be driven by single RNA Pol-III promoter and Cas9 may be expressed from CaMV-35S promoter followed by a NOS terminator sequence. Moreover, each sgRNA should be followed by a DR sequence to facilitate homologous recombination (**Figure 5B**). Finally, the designed cassette could be cloned directly into a binary vector to facilitate *Agrobacterium*-mediated stable genetic transformation in the model plant *N. benthamiana* (**Figure 5C**). The transgenic plants harboring the sgRNA multiplex cassette along with all necessary controls would be challenged by different CABs with and without their cognate DNA-satellites using agroinfiltration (**Figure 5D**). The control plants will start showing typical CLCuD symptoms after 2–3 weeks of post inoculation. Whereas, the transgenic plants harboring sgRNA multiplex cassette would be symptom free depending upon the level of expression of each sgRNA (**Figure 5E**). Once proof-of-concept established in *N. benthamiana* plants then whole system could be tested in the local cotton germplasm such as ‘Cocker’, which is not only susceptible to CLCuD but also easy to transform (Ikram-Ul-Haq, 2004; Sohrab et al., 2014). The plant transformation will be done by *Agrobacterium*-mediated transformation and the fully established transgenic plants will be challenged by viruliferous whiteflies either in the cages or directly exposed to the viral inoculum in the field. The multiplex CRISPR/Cas9 strategy proposed here, could provide a comprehensive approach to control CABs and their associated DNA-satellites simultaneously.

## Conclusion

Development of resistance against CLCuD is an intricate practice amenable to be overcome by counter strategies adopted by either the CABs or associated DNA-satellites. Offering resistance through conventional as well as other synthetic biology approaches is genetically tractable through molecular genetics but the history showed that the resistance, once achieved, was not long-lasting. Alternatively, we have to devise a multifaceted strategy, which could provide unusually precise and specific approach to counter CLCuD-complex. CRISPR/Cas9 system offers a flexible approach for stacking multiple nucleases as one transgene, thereby offering targeted cleavage of mixed infections by multiple viruses and associated DNA-satellites, such as CLCuD-complex. The CRISPR/Cas9 system could be an answer to open up unprecedented possibilities to develop CLCuD resistant cotton plants. Thus, it could be beneficial to the scientific community in devising future resistance strategies against whole CLCuD complex and this approach will be an outstanding example.

## Author Contributions

ZI conceived the idea, performed the bioinformatics analysis for designed sgRNAs and drafted the first draft of the manuscript. MNS designed all the sgRNAs, draw figures and helped in preparation of first draft of the manuscript. MS drew all tables, searched all the web based tools and helped in preparation of first draft of the manuscript. First draft of the manuscript was edited and approved by all authors.

## Conflict of Interest Statement

The authors declare that the research was conducted in the absence of any commercial or financial relationships that could be construed as a potential conflict of interest.
