# Supplementary material for: CRISPR/Cas9: A Tool to Circumscribe Cotton Leaf Curl Disease
Source: Front Plant Sci. 2016 Apr 12;7:475. doi: 10.3389/fpls.2016.00475 (PMC4828465; doi:10.3389/fpls.2016.00475)
Supplement: Supplementary file 1 [file Data_Sheet_1.DOCX]

**Supplementary Table 1:** Analysis of designed sgRNAs against CABs, betasatellite and alphasatellite.

| CABs | | | | | | |
| --- | --- | --- | --- | --- | --- | --- |
| Sr. # | **Target sequence** | **Nucleotide position in genome** | **Off Targets** | ***Specificity based on Score** | | **^1^Viral genomes showing homology with designed sgRNAs** |
| 1. | ATCCGTATAATATTACCGGATGG | 2721-06 (IR) | 11 off targets site in Arabidopsis – none in coding seq. | **97 (0.6)** | | TYLCV, CLCuKoV-Bu, CrYVV, PepLCV, TYLCSV |
|  |  |  |  |  | |  |
| 2. | TTCCCCTGTGCGTGAATCCATGG | 1431-1451 (REn/TrAp) | 09 off targets site in Arabidopsis – none in coding seq. | **98 (0.4)** | | TYLCV, PaLCuV, CLCuKoV-Bu, CLCuMuV, EACMV |
|  |  |  |  |  | |  |
| 3. | TGTGCGTGAATCCATGGTTGTGG | 1437-1454 (REn/TrAp) | 12 off targets site in Arabidopsis – none in coding seq. | **96 (0.3)** | | CLCuMuV, PaLCuV, CLCuKoV-Bu, EACMV |
|  |  |  |  |  | |  |
| 4. | ATCCCGCCTTTAATTTGAACTGG | 1695-1715 (Rep) | 10 off targets site in Arabidopsis – none in coding seq. | **98 (0.4)** | | CLCuBaV, CLCuKoV-Bu, CLCuMuV, PepGMV |
|  |  |  |  |  | |  |
| 5. | CCTGTGCGTGAATCCATGGTTGT | 1458-1438 (REn/TrAp) | 05 off targets site in Arabidopsis – none in coding seq. | **99 (0.3)** | | PaLCuV, CLCuKoV-Bu, CLCuMuV, SLCMV, AEV, EACMV, TYLCV |
|  |  |  |  |  | |  |
| 6. | CCTTTAATTTGAACTGGCTTCCC | 1724-1704 (Rep) | 10 off targets site in Arabidopsis – none in coding seq. | **97 (1.2) not good** | | SPLCuV, OELCuV, BhYVV, CLCuMuV, CLCuKoV-Bu, SLCMV, |
|  |  |  |  |  | |  |
| 7. | CCTTCGAACTGGATGAGAACATG | 2411-2391 (Rep/C4) | 16 off targets site in Arabidopsis – none in coding seq. | **97 (0.8)** | | CLCuMuV, HoLCV, OEV, CLCuKoV-Bu, BhYVV |
|  |  |  |  |  | |  |
| 8. | CCATTGTCCGCGTCACCAAAGCA | 400-380 (Cp/Pre-Cp) | 06 off targets site in Arabidopsis – none in coding seq. | **98 (0.5)** | | ToLCGuV, CLCuKoV-Bu, ToLCKnV, PaLCuV, AEV |
|  |  |  |  |  | |  |
| 9. | CCATGGTTGTGGCAGTTGATTGA | 1471-1451 (REn/TrAp) | 18 off targets site in Arabidopsis – none in coding seq. | **97 (0.4)** | | CLCuMuV, CLCuRaV, CLCuKoV-Bu |
|  |  |  |  |  | |  |
| 10. | CCGGATGGCCGCGCGATTTTTT | 23-3 (IR) | 01 off targets site in Arabidopsis – none in coding seq. | **99 (0.0) very good** | | CLCuKoV-Bu. OELCV, BhYVV |
|  |  |  |  |  | |  |
| 11. | CCGCGCGATTTTTTTGTGGGCCC | 32-12 (IR) | 03 off targets site in Arabidopsis – none in coding seq. | **98 (0.3)** | | CLCuKoV-Bu, CLCuMuV, CLCuKoV-Sha, CLCuRaV |
|  |  |  |  |  | |  |
| 12. | CCGTCGATCTGAAATTGCCCCCA | 2249-2229 (Rep/C4) | 07 off targets site in Arabidopsis – none in coding seq. | **98 (1.0)** | | CLCuMuV, CLCuRaV, CLCuKoV-Bu, CLCuKoV |
|  |  |  |  |  | |  |
| 13. | CCCATTGTCCGCGTCACCAAAGC | 399-379 (Cp/Pre-cp) | 07 off targets site in Arabidopsis – none in coding seq. | **98 (0.4)** | | ToLCGuV, CLCuKoV-Bu, ToLCKnV, PaLCuV, AEV |
|  |  |  |  |  | |  |
| 14. | CCCCTGTGCGTGAATCCATGGTT | 1456-1436 (REn/TrAp) | 12 off targets site in Arabidopsis – none in coding seq. | **98 (0.4)** | | TYLCV, PaLCuV, CLCuKoV-Bu, SLCMV, EACMV |
|  |  |  |  |  | |  |
| 15. | CCCTGTGCGTGAATCCATGGTTG | 1457-1437 (REn/TrAp) | 07 off targets site in Arabidopsis – none in coding seq. | **98 (0.4)** | | CLCuKoV-Bu, CLCuMuV, TYLCV, PaCrV, HoYVMV. AEV, |
|  |  |  |  |  | |  |
| 16. | CCCTCGAACTGGATGAGCACATG | 2411-2391 (Rep/C4) | 03 off targets site in Arabidopsis – none in coding seq. | **99 (0.1)** | | CLCuMuV, BhYVV, CLCuKoV-Bu, ToLCV |
| Alphasatellite | | | | | | |
| Sr. # | **Target sequence** | **Nucleotide position in genome** | **Off Targets** | **Specificity based on Score** | **^2^Alphasatellite genomes showing homology with designed sgRNAs** | |
| 1. | AATTCGAAGTCCGGTGAGAATGG | 437-458 (Rep) | 11 off targets site in Arabidopsis – none in coding seq. | **98 (0.4)** | AgLCA, ChLCA, CLCuMuA, CLCuBuA, | |
|  |  |  |  |  |  | |
| 2. | CAGATGACCGCACTATCTTCTGG | 600-619 (Rep) | 03 off targets site in Arabidopsis – none in coding seq. | **99 (0.3)** | AgLCA, ChLCA, CLCuMuA, CLCuBuA | |
|  |  |  |  |  |  | |
| 3. | AGATGACCGCACTATCTTCTGGG | 601-620 (Rep) | 06 off targets site in Arabidopsis – none in coding seq. | **98 (0.3)** | AgCSLA, CLCuMuA, SiLCA, GuLCA, OELCA | |
|  |  |  |  |  |  | |
| 4. | CCTGCTGGCAGGAAGAAGAGTCG | 180-199 (Rep) | 07 off targets site in Arabidopsis – none in coding seq. | **98 (0.6)** | CLCuMuA, CLCuBuA, AgCALA, OELCA | |
|  |  |  |  |  |  | |
| 5. | CCTGGTTCTATACACGTGGAGGA | 709-690 (Rep) | 05 off targets site in Arabidopsis – none in coding seq. | **97 (1.1)** | CLCuMuA, CLCuBuA, AgCSLA | |
|  |  |  |  |  |  | |
| 6. | CCAGATGACCGCACTATCTTCTG | 621-602 (Rep) | 06 off targets site in Arabidopsis – none in coding seq. | **99 (0.3)** | CLCuMuA, SiLCA, GuLCA, OELCA | |
|  |  |  |  |  |  | |
| 7. | CCGAGGCTGGTGTAGTATTACCC | 1364-4 (IR) | 02 off targets site in Arabidopsis – none in coding seq | 99 (0.2) | CrYVA, BhYMA, RadLCA, CLCuMuA, CLCuBuA | |
|  |  |  |  |  |  | |
| 8. | CCCAGATGACCGCACTATCTTCT | 620-601 (Rep) | 10 off targets site in Arabidopsis – none in coding seq. | **98 (0.3)** | CLCuMuA, CLCuBuA, AgCALA, SiYVMA, | |
| Betasatellite | | | | | | |
| Sr. # | **Target sequence** | **Nucleotide position in genome** | **Off targets** | **Specificity based on Score** | **^3^Betasatellite genomes showing homology with designed sgRNAs** | |
| 1. | ACATCCATTCCCAATATCTCTGG | 235-254 (βC1) | 14 off targets site in Arabidopsis – none in coding seq. | **95 (1.3)** | ChLCB, CLCuMuB, OkLCB, MalYVB, | |
|  |  |  |  |  |  | |
| 2. | GTGTGTACCCCTGGGAGGGTAGG | 1265-1284 | 03 off targets site in Arabidopsis – none in coding seq. | **99 (0.3)** | CLCuMuB, ToLCB, OELCB, BhYVB, LuLB, ChLCB | |
|  |  |  |  |  |  | |
| 3. | TTAATATTACCGTGGGCGAGCGG | 1343-12 (IR) | 03 off targets site in Arabidopsis – none in coding seq. | **99 (0.1)** | CLCuMuB, OkLCB, ChLCB, MalYVB | |
|  |  |  |  |  |  | |
| 4. | CGCAGCAGCCTTAGCTACGCCGG | 1301-1320 | 05 off targets site in Arabidopsis – none in coding seq. | **98 (0.5)** | CLCuMuB, TbLCB, CLCuBuB, OkLCB, BhYVB, | |
|  |  |  |  |  |  | |
| 5. | CCACGTTCTAATATTACCGTGGG | 1336-5 (IR) | 01 off targets site in Arabidopsis – none in coding seq. | **99 (0.8)** | CLCuMuB, CLCuBuB, OkLCB, MalYVB ChLCB, | |
|  |  |  |  |  |  | |
| 6. | CATCCATTCCCAATATCTCTGGG | 236-255 (βC1) | 20 off targets site in Arabidopsis – none in coding seq. | **93 (1.3)** | CLCuMuB, CLCuBuB | |
|  |  |  |  |  |  | |
| 7. | GGCGGTGTGTACCCCTGGGAGGG | 1261-1280 | 01 off targets site in Arabidopsis – none in coding seq. | **99 (0.1)** | CLCuMuB, CLCuBuB, BhYVB, OkLCB, ToLCB, ChLCB, | |
|  |  |  |  |  |  | |
| 8. | CCTTCAAAGCCGTTGAAGTCGAA | 383-364 (βC1) | 16 off targets site in Arabidopsis – none in coding seq. | **97(0.7)** | CLCuMuB, CLCuBuB, LuLDB | |
|  |  |  |  |  |  | |
| 9. | CCTCATGTGAATGAAGATCTTCA | 484-463 (βC1) | 35 off targets site in Arabidopsis – none in coding seq. | **93(0.8)** | CLCuMuB, BhYVB, OkB, ToLCB, ChLCB, | |
|  |  |  |  |  |  | |
| 10. | CCATTCCCAATATCTCTGGGTTT | 261-242 (βC1) | 19 off targets site in Arabidopsis – none in coding seq. | **96(0.8)** | CLCuMuB, CLCuMuB, LdLDB, LuLCB, | |
|  |  |  |  |  |  | |
| 11. | CCAATATCTCTGGGTTTTCAAGT | 267-248 (βC1) | 20 off targets site in Arabidopsis – none in coding seq. | **96 (0.9)** | CLCuMuB, LdLDB, LuLCB, | |
|  |  |  |  |  |  | |
| 12. | CCCAATATCTCTGGGTTTTCAAG | 266-247 (βC1) | 22 off targets site in Arabidopsis – none in coding seq. | **95 (0.7)** | CLCuMuB, CLCuBuB, LdLDB, LuLCB, | |

*All sgRNAs are scored by inverse likelihood of off-target binding against *A. thaliana* genome. Numbers written within the parenthesis are showing inverse likelihood. ([www.crispr.mit.edu](http://www.crispr.mit.edu)).

The PAM sequence of each sgRNA is indicated in Red, whereas mismatches outside the seed sequence (shown in blue color – first 12 nucleotides at upstream of PAM) are highlighted in white text on a black background

**^1^Abbreviations of viruses and satellites showing homology with designed sgRNAs** (All the names are according to Brown et al., 2015):

BhYVV=Bhindi yellow vein virus

CLCuBuV=Cotton leaf curl Kokhran virus-Burewala

CLCuKoV=Cotton leaf curl Kokhran virus

CLCuKoV-Sha=Cotton leaf curl Kokhran virus-Shahdadpur

CLCuMuV=Cotton leaf curl Multan virus

CLCuRaV=Cotton leaf curl Rajasthan virus

CrYVV=Croton yellow vein virus

EACMV=East African cassava mosaic virus

HoLCV=Hollyhock leaf curl virus

OELCuV=Okra enation leaf curl virus

OEV=Okra enation virus

PaCrV=Papaya crumple virus

PaLCuV=Papaya leaf curl virus

PepGMV=Pepper golden mosaic virus

PepLCV=Pepper leaf curl virus

SLCMV=Sri Lankan cassava mosaic virus

SPLCV=Sweetpotato leaf curl virus

ToLCGuV=Tomato leaf curl Gujrat virus

ToLCKnV=Tomato leaf curl Karnatka virus

ToLCNDV=Tomato leaf curl New Delhi virus

TYLCSV= Tomato yellow leaf curl Sardinia virus

TYLCV=Tomato yellow leaf curl virus

**^2^ Abbreviations of alphasatellites with their full names:**

AgCSLA=Ageratum conyzide symptomless alphasatellite

BhYMA=Bhindi yellow mosaic alphasatellite

BhYVMA=Bhindi yellow vein mosaic alphasatellite

CLCuBuA= Cotton leaf curl Burewala alphasatellite

CLCuMuA=Cotton leaf curl Multan alphasatellite

CrYVA=Croton yellow vein alphasatellite

GuLCA=Guar leaf curl alphasatellite

OELCA=Okra enation leaf curl alphasatellite

RadLCA=Radish leaf curl alphasatellite

SiLCA=Sida leaf curl alphasatellite

SiLCA=Sida leaf curl alphasatellite

**^3^ Abbreviations of betasatellites with their full names:**

BhYVB=Bhindi yellow vein betasatellite

ChLCB=Chilli leaf curl betasatellite

ChLCB=Chilli leaf curl betasatellite

CLCuBuB= Cotton leaf curl Burewala betasatellite

CLCuMuB=Cotton leaf curl Multan betasatellite

LdLDB= Ludwigia leaf distortation betasatellite

LuLCB=Lulliby leaf curl betasatellite

MalYVB =Malvastrum yellow vein betasatellite

OkLCB=Okra leaf curl betasatellite

ToLCB= Tobacco leaf curl betasatellite

ToLCB=Tobacco leaf curl betasatellite
